# Experimental Research and Analysis on Fatigue Life of Carbon Fiber Reinforced Polymer (CFRP) Tendons

**DOI:** 10.3390/ma12203383

**Published:** 2019-10-16

**Authors:** Shoutan Song, Hua Zang, Ning Duan, Juntao Jiang

**Affiliations:** 1 School of Civil Engineering, Southeast University, Nanjing 210096, China; duan_n@outlook.com (N.D.); 220171055@seu.edu.cn (J.J.); 2 Key Laboratory of Concrete and Prestressed Concrete Structures Ministry of Education, Southeast University, Nanjing 210096, China; zanghua2003@163.com; 3 School of Architecture Engineering, Nanjing Institute of Technology, Nanjing 211167, China

**Keywords:** carbon fiber reinforced polymer (CFRP), fatigue life, stress range, maximum stress, reliability analysis

## Abstract

The fatigue life of carbon fiber reinforced polymer (CFRP) tendons was studied in this paper. A new wedge-type anchorage system was applied to the fatigue test of CFRP tendons and demonstrated an excellent fatigue resistance. In the test and analytical data, the fatigue stress ranged from 200 MPa to 800 MPa, and maximum stresses from 0.37 to 1.0 $f_{u}$ ($f_{u}$ = ultimate tensile strength of CFRP tendons) were determined. The main work and results were that the stress range and stress level (maximum stress) were two key parameters affecting the fatigue life of CFRP tendons. A bilinear equation and a linear equation considering the fatigue life of CFRP tendons jointly affected by the stress range and the maximum stress were established. The error of predicted results and test results was 0.038 and 0.083, respectively, both representing good prediction accuracy. The predicted results of Whitney’s method showed that, at a 95% confidence level, when the stress range was 200 MPa, 400 MPa, and 600 MPa, the maximum stress limit of CFRP tendons, which were not broken in a fatigue test of 2 million times, was 63.9% $f_{u}$, 53.0% $f_{u}$, and 36.8% $f_{u}$, respectively.

## 1. Introduction

Fiber-reinforced polymer (FRP) composites have the characteristics of light weight, high strength, corrosion resistance, and electromagnetic insulation, which can replace steel for engineering construction in particular scenarios [1,2,3]. On the basis of the fiber variety, FRP can be classified into glass fiber-reinforced polymer (GFRP), carbon fiber-reinforced polymer (CFRP), basalt fiber-reinforced polymer (BFRP), and aramid fiber-reinforced polymer (CFRP), among others. Among all FRPs, CFRP has the properties of superior fatigue resistance [4,5,6,7], high-creep rupture limit [8,9,10], low-creep performance [11], and other benefits, which has deemed it as the most ideal cable material to replace steel, thereby realizing the long life of major engineering structures. Cable structures bear repetitive loads during their service life. Fatigue rapture may bring disastrous consequences due to its burstiness. Therefore, a study on the high-precision prediction method of fatigue life of CFRP cables is required.

Stable and reliable anchorages are key to applying FRP cables to practical application. Due to low transverse strength and the delicate surface of FRP tendons, a dedicated anchorage system for FRP cables needs to be developed. The authors’ research team developed a new wedge-type anchorage system and in [11] applied this new device to the study of long-term creep performance of CFRP tendons. This paper applied this anchorage system to the fatigue performance test of the pre-stressed CFRP tendons. The fatigue stability of the anchorage system was also tested.

Maximum stress $\sigma_{max}$ and minimum stress $\sigma_{min}$ are two parameters that need to be set in the fatigue performance test of CFRP tendons, which can be used to obtain stress range Δσ and stress ratio R. Saadatmanesh and Tannous [12] studied the effects of the stress range and the maximum stress on the fatigue performance of CFRP tendons. The experimental results suggested that for the stress range of 100 MPa, 200 MPa, and 400 MPa, the fatigue lives of CFRP tendons were larger than 3 million cycles when the maximum stresses were lower than 1900 MPa, 1400 MPa, and 1000 MPa, respectively. Adimi et al. [13] studied the fatigue life of CFRP tendons when the stress ratio was 0.1. The results showed that the fatigue life increased linearly with the decrease of the maximum stress in the CFRP tendons. For the specimen subjected to a maximum stress lower than 1000 MPa, the fatigue life was longer than 4 million cycles. Zhang et al. [14] compared the fatigue behavior of CFRP tendons for the stress ratios of 0.0 and 0.5. The fatigue life was demonstrated to increase with the larger stress ratios. Adimi et al. [13] also pointed out that the ambient temperature and loading frequency may affect the fatigue life of CFRP tendons. The above experiments demonstrated that the fatigue life of CFRP tendons are closely related to stress level (maximum stress or minimum stress) and the stress range. 

The fatigue life of CFRP tendons can be predicted by establishing S-N curve with limited fatigue test data. Adimi et al. [13] plotted the linear relationship S-N curve of $\log\sigma_{max}$ and logN with the stress ratio of CFRP tendons at 0.1, whereas Zhang et al. [14] plotted the exponential relationship S-N curve of $\sigma_{max}$ and logN with the stress ratio of CFRP tendons at 0.5. Wu et al. [7] plotted the linear relationship S-N curve of $\sigma_{max}$ and logN with the stress ratio of CFRP sheets at 0.1. Feng et al. [15] used the linear relationship S-N curve of $\sigma_{max}$ and logN to evaluate the fatigue life of CFRP cables. The experimental *S-*N curves obtained from the above references showed evident variations because the CFRP specimens tested varied in types (tendons or sheets), fiber types, and fiber volume content. The stress level parameter on S-N curve is one of $\log\sigma_{max}$, $\sigma_{max},$ or Δσ, with different forms, and S-N curves derived from data points of individual stress ratio cannot fully reflect the fatigue performance of CFRP materials.

In practical applications, the maximum stress of FRP is limited to avoid the fatigue life of FRP. The limited maximum stress is determined on the basis of the *S*-N curve and the probabilistic analysis. At present, three methods are commonly used for performing probabilistic analysis of FRP’s fatigue life: normal lifetime distribution (NLD) method [16], American Society for Testing and Materials E739-10 method (ASTM 2010) [17], and Whitney’s method [16]. NLD method is based on the normal distribution and can be readily used without the need for iteration. Whitney’s method is based on the Weibull distribution. Although it can reflect the variations in the material properties of FRP, an iteration computing procedure is required [18]. ASTM method is only applicable in cases where the linear material behavior hypothesis in terms of the *S-*N curve is confirmed.

The anchoring types of FRP tendons are mainly divided into bonded anchorage and mechanical anchorage. Bonded anchorage is characterized by longer anchoring length and complex process. Adimi et al. [13] anchored the CFRP tendons into concrete with the anchoring length reaching 250 mm. Wang et al. [19] radially wound a bidirectional basalt fiber sheet along tendons at BFRP tendon anchorage and solidified it to form a diameter enlargement area for loading the wedge. The experiments performed by Xie et al. [20] suggest that the stress range affects the slippage of a bonded anchorage system. When the maximum fatigue load reaches 50.6% of the ultimate strength of CFRP tendons and the stress range is lower than 10.6% of the ultimate strength of the tendons, the increase of the stress range may lead to the fatigue failure of the anchorage system. Mechanical anchorage can be conducted in multiple types, such as split-wedge anchorage, nonmetallic wedge anchorage, and integrated sleeve-wedge anchorage [21]. It has a greater advantage of assembly efficiency. Due to the low compressive strength of FRP, the compressive force perpendicular to the tendon resulted from mechanical anchorage tends to cause fracture at the anchorage of FRP tendons. To overcome this problem, some researchers [12,22,23] placed a flexible sleeve between the wedge and tendons to distribute the compressive stresses. Sayed-Ahmed [22] and Al-Mayah [24] proposed the differential angle design and curved angle design, respectively, which can transfer the compressive stresses to the back of the anchorage where the tensile stresses are lower. However, the stability, reliability, and economic performance of these mechanical anchorages still need to be improved [21]. The development of a stable and efficient anchorage for FRP tendons is an important task in the application study of CFRP cables.

On the basis of the new wedge-type anchorage of FRP tendons developed by the research team, this paper conducted an experimental study on the fatigue life of CFRP tendons. In the test, the stress range Δσ was 600 Mpa and 800 Mpa, and the maximum stress ranged from 0.375 to 0.843 $f_{u}$. On the basis of the test data of this paper and that in [12], a bilinear model, which considers the fatigue life of CFRP tendons jointly affected by stress range and maximum stress, was established. Meanwhile, the reliability of the fatigue life of CFRP tendons was analyzed.

## 2. Materials and Methods

### 2.1. Characteristics and Anchorages of CFRP Tendons

Smooth and round CFRP tendons with a diameter of 8 mm produced in Jiangsu Hengshen (Zhenjiang, China) were employed in this study. The tendons were made from 12k, Type T700 continuous CFRP filaments (Hengshen, Zhenjiang, China), which were immersed in high toughness epoxy resin and then solidified, having a volume content of 65% and a density of 1.6 g/cm^3^. 

**Figure 1:**
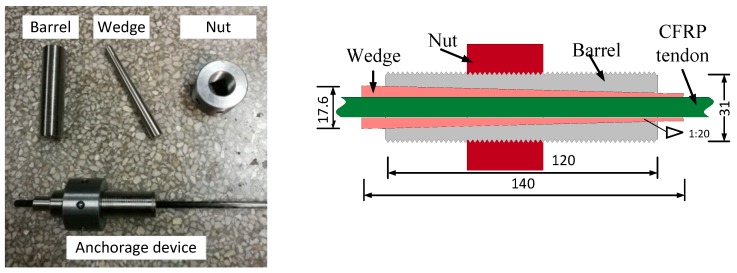
Wedge-type anchorage for the test. CFRP: carbon fiber reinforced polymer. (Units: mm)

A new anchorage system developed by the research team is shown in Figure 1, which mainly included the steel wedge, barrel, and nut. To reduce the friction between the wedge and the barrel, the wedge’s outer surface was polished and the taper angle was made 1:20. The wedge was manufactured with a flexible thin layer on its inner surface. In this manner, the placement of an additionally flexible sleeve, which is commonly used at present [24,25,26], was omitted, such that the assembly was simplified. The incorporation of the flexible thin layer not only increased the friction between the wedge and the CFRP tendons, but also avoided the premature failure of FRP tendons due to the concentrated stress induced by the clamp load [27]. The nut was connected with the barrel. In practical applications, the nut was placed against the anchorage plates fixed on the end of the concrete member to counteract the load caused by prestressing the tendons.

### 2.2. Testing Setups

#### 2.2.1. Static Test Setup

**Figure 2:**
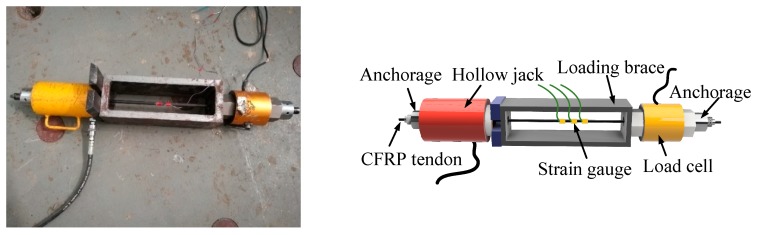
Static test setup.

The static tensile strength test system was used to test the static strength and static elastic modulus of CFRP tendons under static conditions. As shown in Figure 2, the system mainly consisted of a hollow jack, a load cell, and a loading brace. The loading brace was employed to observe the rupture of CFRP tendons. A strain gauge was placed in the middle of the CFRP tendons to record the tendon strain during the loading process. The load cell was used to record the external load, thereby determining the stress in the tendons. A sliding tag was set on the surface of CFRP tendons near the anchorage area to observe the slippage of tendons.

#### 2.2.2. Fatigue Test Setup

**Figure 3:**
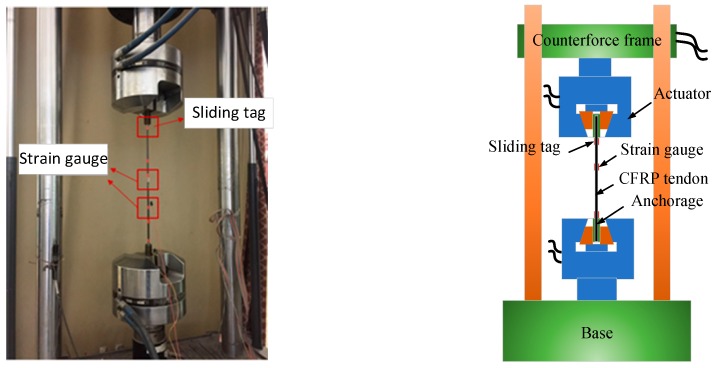
Fatigue test setup.

The fatigue performance test of CFRP tendons was performed using the MTS-810 fatigue testing machine. The test loading was controlled by load, with the accuracy of 0.1 kN. The specialized wedge of the testing machine directly clamped the cup in the anchorage to exert fatigue load, as shown in Figure 3. The free length of CFRP tendons was 500 mm. A mark was set on the surface of CFRP tendons near the interior of the anchorage area to observe the slippage between CFRP tendons and the wedge during fatigue loading. A strain gauge was installed in the middle of the tendons to record the tendon strain.

### 2.3. Loading Procedure

#### 2.3.1. Static Test Loading Procedure

The loading rate was set as 200 MPa/min in the static strength test, and the load and tendon strain were recorded by TDS-530 static data acquisition machine (Tokyo institute of instrumentation, Tokyo, Japan) at the frequency of 1 Hz. The static elastic modulus of CFRP tendons was obtained from the data points of ultimate tensile strength at 20% and 50%.

#### 2.3.2. Fatigue Test Loading Procedure

**Figure 4:**
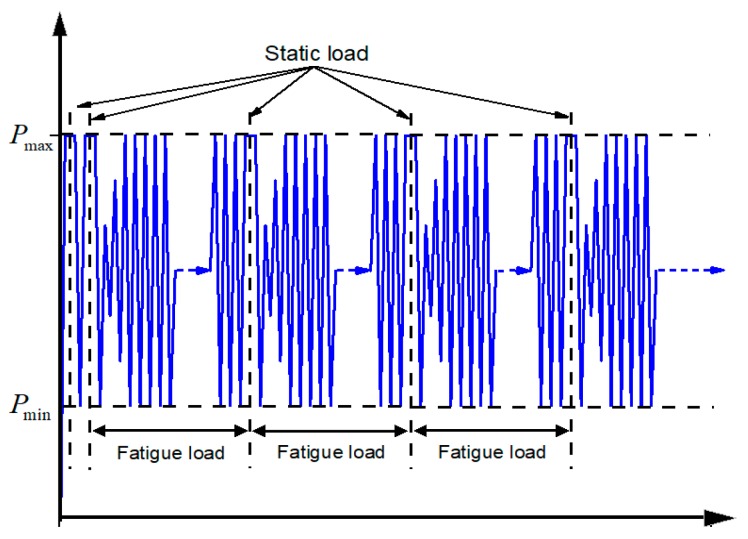
Loading mode.

The whole process of this fatigue test adopted the loading method of constant amplitude sinusoid. The loading frequency in this paper was 5 Hz. The study [28] showed that the temperature rise inside the materials can be ignored when the loading frequency is less than 10 Hz. The fatigue loading was divided into the following stages: static pre-loading, twice-static loading, followed by fatigue loading. The upper limit of the fatigue exerted by the pre-loading was 20% of the load, with the purpose of removing the spaces and poor contact between the wedges. The twice-static loading stage was done to record the initial elastic modulus of CFRP tendons. The formal fatigue loading conducted the static loading test during shutdown at fixed times to obtain the residual fatigue elastic modulus. The loading mode was shown in Figure 4. This paper focused on the fatigue life of CFRP tendons and, hence, the residual elastic modulus was not included. When tendons were ruptured with the fatigue cycle being less than 2 million, the cycles at rupture were recorded. When tendons were not ruptured with the fatigue cycle reaching 2 million, the static loading continued until rupture occurred and the strength of tendons after fatigue loading was tested.

## 3. Results and Discussion

### 3.1. Static Tensile Properties

**Figure 5:**
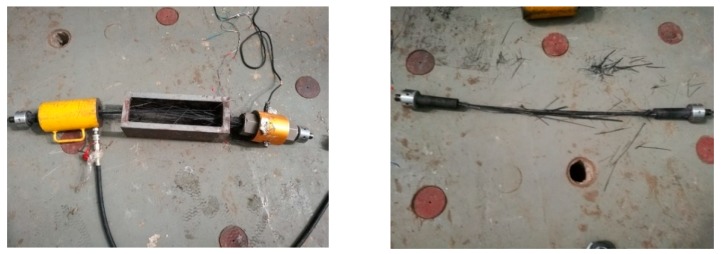
Failure mode of CFRP tendons.

In the static loading test, the five specimens demonstrated the same failure mode, namely, burst fiber rupture in the middle, as shown in Figure 5. No tendon slippage or premature failure was detected in the anchorage area, which indicated the efficiency of the anchorage. The static test data of CFRP tendons are shown in Table 1, where the average static strength was 2136 MPa (Coefficient of variation, COV, 3.2%), and the average static elastic modulus was 150.2 GPa (COV 2.1%). For design consideration, the ultimate tensile strength of CFRP tendons was determined to be 2024 MPa, with a 95% confidence level calculated using the Equation (1):

(1) $$f_{k}=\mu_{f}-1.645\sigma_{f}$$

where $f_{k}$, $\mu_{f},\sigma_{f}$ are the strength standard value, average value, and variance of CFRP tendons, respectively.

### 3.2. Fatigue Tensile Properties

**Figure 6:**
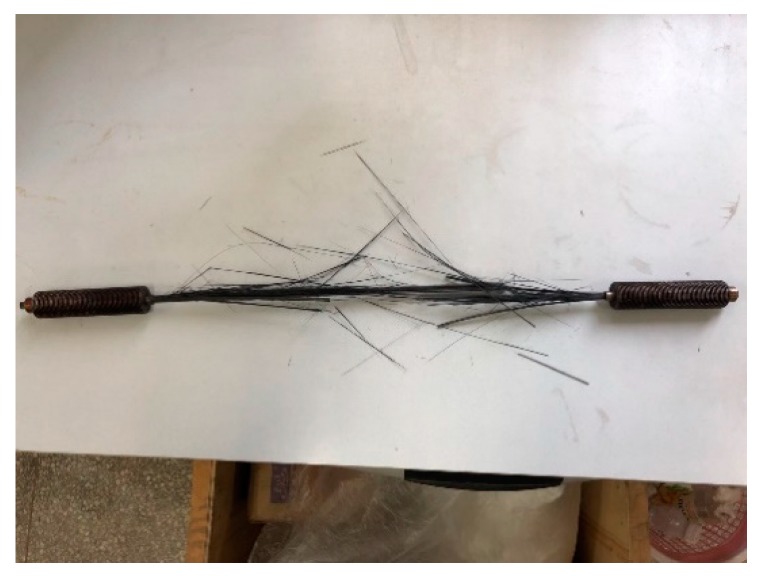
Fatigue failure of tendons.

The failure mode of CFRP tendons is shown in Figure 6. All of the specimens exhibited a similar failure mode in the middle portion. There was no slip and advance failure in the anchorage area, which indicated that the anchorage had good fatigue stability. The fatigue life data of each specimen are shown in Table 2.

As seen from the data in Table 2, at the same stress range, the fatigue life of CFRP tendons increased with decreasing maximum stress. At the stress range of 600 MPa, the average fatigue life of CFRP tendons was 541 cycles with the maximum stress of 1800 MPa, whereas the fatigue life of CFRP tendons exceeded 2 million cycles with the maximum stress reduced to 800 MPa. After 2 million cycles of fatigue loading for specimen 16, the static strength of CFRP tendons was 2081 MPa, about 97.4% of the average static strength of CFRP tendons. At the same maximum stress, the fatigue life of CFRP tendons with the stress range of 800 MPa was 11.7–41.0% of that with the stress range of 600 MPa. Evidently, the stress range and the maximum stress jointly affected the fatigue life of CFRP tendons.

### 3.3. Discussion

**Figure 7:**
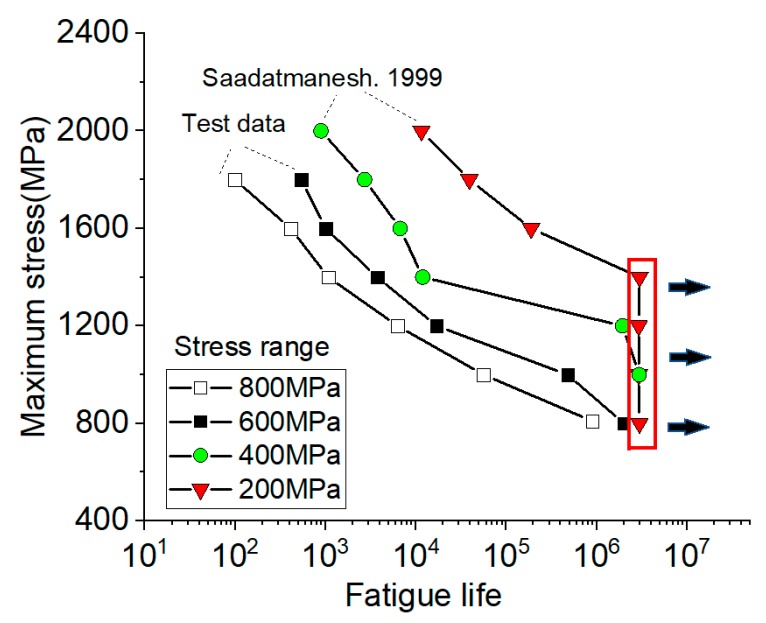
Test data.

**Figure 8:**
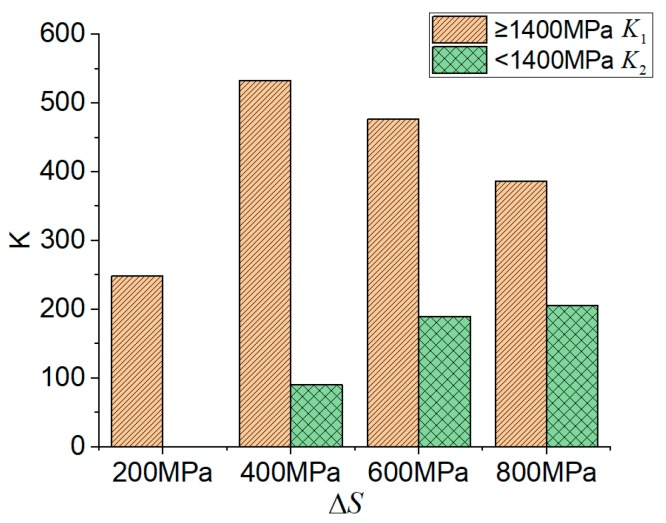
Rate of fatigue life.

The fatigue life data of CFRP tendons with the stress ranges of 200 MPa, 400 MPa, 600 MPa, and 800 MPa are shown in Figure 7, where the stress ranges of 200 MPa and 400 MPa are from [12], and 600 MPa and 800 MPa are the test results of this paper. In [12], leadline tendons were fabricated with PAN-type carbon fibers (fiber volume fraction = 65%) embedded in an epoxy matrix. The ultimate strength was 1999.2 MPa (COV 1.6%). The static elastic modulus was 149.6 GPa (COV 8.3%). The material properties of the tendons reported by [12], including the fiber type, fiber volume fraction, and the ultimate strength, were similar to the tendons of this paper, which makes the experimental results of these two groups comparable. The data points in Figure 8 represent the average value of several test results at the same level. As can be seen from these data, the stress range and the stress level (the maximum stress) jointly affected the fatigue life of CFRP tendons. At the same maximum stress, the greater the stress range, the shorter the fatigue life of CFRP tendons. At the same stress range, the fatigue life of CFRP tendons increased with the lowering of the maximum stress. The changing speed of fatigue life varied at different stages, which was slow with greater maximum stress.

Coefficients $K_{1}$ and $K_{2}$ were adopted in this paper to separately describe the changing rate of fatigue life for the case where $\sigma_{max}$ was greater than 1400 MPa and those where $\sigma_{max}$ was smaller than 1400 MPa. Equation (2) is shown below:

(2) $$K_{1}=\left|\frac{\sigma_{max1}-\sigma_{1400}}{\log N_{1}-\log N_{1400}}\right|,\;K_{2}=\left|\frac{\sigma_{1400}-\sigma_{max2}}{\log N_{1400}-\log N_{2}}\right|,$$

where $\sigma_{max1}$ and $\sigma_{max2}$ represent the maximum and minimum $\sigma_{max}$ on the same stress range curve. At the stress ranges of 200 MPa, 400 MPa, 600 MPa, and 800 MPa, $\sigma_{max1}$ was 2000 MPa, 2000 MPa, 1800 MPa, 1800 MPa, respectively, and $\sigma_{max2}$ was 1400 MPa, 1200 MPa, 1000 MPa, and 800 MPa, respectively; $\sigma_{1400}$ = 1400 MPa; $N_{1}$ and $N_{2}$ are the fatigue life at $\sigma_{max1}$ and $\sigma_{max2}$; $N_{1400}$ is the fatigue life when $\sigma_{max}$ = 1400 MPa.

The calculation results of $K_{1}$ and $K_{2}$ at different stress ranges are shown in Figure 8 (Supplementary material Table S1). It can be seen that $K_{2}$ was considerably less than $K_{1}$ at the same stress range, and the difference between $K_{1}$ and $K_{2}$ was smaller with increasing stress range. At the stress range of 400 MPa, *S*-N curve of the maximum stress $\sigma_{max}$ and fatigue life (logN) presented the form of double broken lines. With increasing stress range, *S*-N curve tended to display a linear relationship. This figure reflects that different stress ranges brought different changing rates of fatigue life.

As shown in Figure 8, the effect of the stress range on the slope *K* of *S*-N curves was evident. In addition, the slope *K* varied with the maximum stress. Most of the available methods for predicting the *S*-N curve ignore the effect of the stress range. To take into account both the effect of the stress range and the varying slopes of the *S*-N curve with the maximum stress, a bilinear equation, which has the simplest form for practical application, is proposed for predicting the fatigue life of CFRP tendons in Equation (3):

(3) $$\sigma_{max}=a+b\cdot \Delta \sigma +(c+d\cdot \Delta \sigma )\cdot \log N.$$

There are four parameters in this model, namely, *a*, *b*, *c*, *d*, among which parameters *b* and *d* are the effects of stress range Δσ on the fatigue life and its changing speed. At the same time, two stages were divided at $\sigma_{max}$ = 1400 Mpa. The values of the four parameters were determined by fitting the experimental results. If $\sigma_{max}$ was no less than 1400, the optimal values of *a*, *b*, *c*, *d* were 2931, −0.005, −151, −0.47, respectively. If $\sigma_{max}$ was less than 1400, the optimal values of *a*, *b*, *c*, *d* were 1661, 0.42, −7.5, −0.25, respectively.

**Figure 9:**
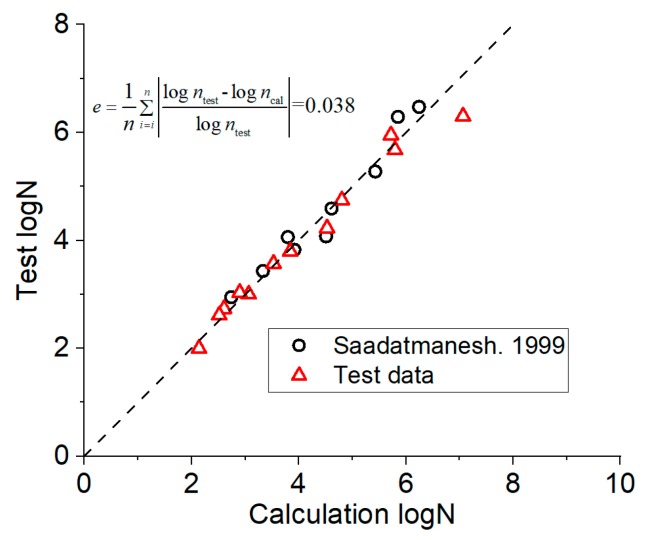
Between calculation results of prediction Equation (3) and test results.

The comparison of the calculation results of prediction in Equation (3) with test results is shown in Figure 9 (Supplementary material Table S2). The test data were mainly derived from the results of [12] and the test in this paper, and the test values corresponding to the data points in the figure are the average values of test results at the same level. The error *e* of the prediction results was 0.040, and prediction Equation (3) has high prediction accuracy. In Figure 9, $n_{test}$ is test data and $n_{cal}$ is the calculated value of Equation (3).

Prediction Equation (3) has higher accuracy in predicting the fatigue life of CFRP tendons, but with slightly complex forms. The stress range Δσ and the maximum stress $\sigma_{max}$ were the main factors influencing the fatigue life of CFRP tendons. A simplified equation to calculate the fatigue life is given in the paper, as shown in Equation (4):

(4) $$\sigma_{max}=a_{1}-b_{1}\cdot \Delta \sigma -c_{1}\cdot \log N.$$

There are three parameters in this model, namely, $a_{1},b_{1},c_{1}$. According to the test results, the optimal values of $a_{1},b_{1},c_{1}$ were 3031, −1.16, and −231, respectively.

**Figure 10:**
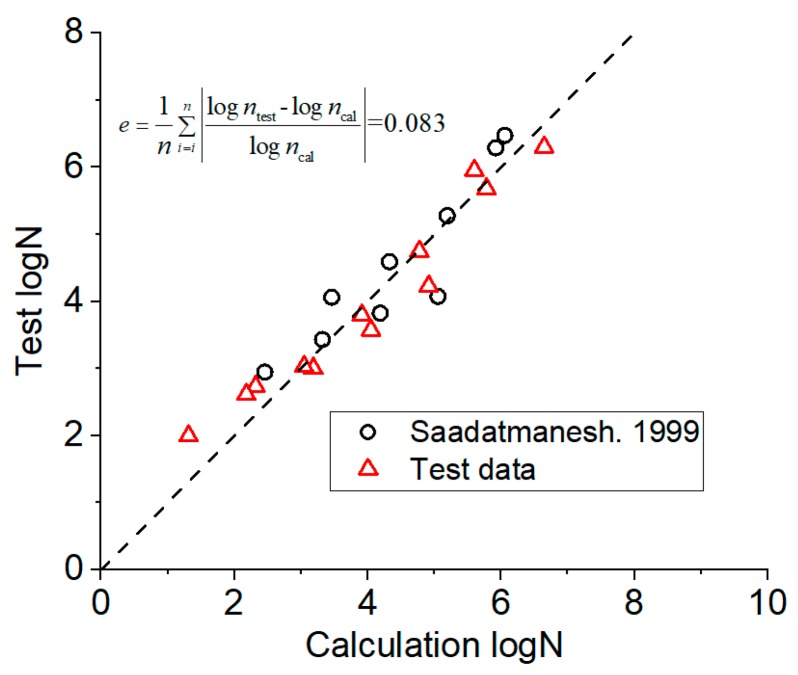
Between calculation results of simplified Equation (4) and test results.

The comparison of the prediction results of Equation (4) with the test results is shown in Figure 10 (Supplementary material Table S3). The error of prediction results was 0.085 and the simplified calculation Equation (4) also had better prediction accuracy. In Figure 10, $n_{test}$ is test data and $n_{cal}$ is the calculated value of Equation (4).

As shown in Table 2, at the same stress level, the fatigue life of CFRP presented relatively great discreteness. A certain reliability should be considered to predict the fatigue life of CFRP tendons during the design. Therefore, on the basis of the test data (from this paper and [12]), the fatigue life reliability of CFRP tendons was analyzed. The normal lifetime distribution (NLD) [16] method and Whitney’s method [16] were used to predict the fatigue life of CFRP tendons.

NLD method is a simplified function model based on probability distribution. It was assumed that the fatigue life of each specimen was in normal distribution and the variation coefficient was 15%. On the basis of the assumption above, the safety guarantee rate was taken as 95%, and the fatigue life at each load level was calculated in Equation (5):

(5) $$R_{ki}(5\%,95\%,15\%,m_{i})={\bar{N}}_{i}\left[1-0.15(1.645+1.645/\sqrt{m_{i}}\right]$$

where $m_{i}$ refers to the number of specimens at the ith load level, and ${\bar{N}}_{i}$ refers to the average fatigue life at the ith load level. 

Through fitting, the *S*-N curve between the load level with 95% guarantee rate and the fatigue life can be obtained, and the curve can be expressed as Equation (6):

(6) $$\sigma =\sigma_{0}\cdot R_{k}^{-1/k}$$

where σ refers to the stress level, referring to the maximum stress in this paper; $R_{k}$ refers to the characteristic value of fatigue life; and k are fitting parameters.

Whitney’s method is based on two assumptions: (1) the *S*-N curve conforms to the power function, i.e., Equation (6); (2) the fatigue life conforms to the two-parameter Weibull distribution, as Equation (7):

(7) $$P_{s}(N_{i})=\exp{\left[-(N_{i}/{\bar{N}}_{i})\right]}^{\alpha_{fi}}$$

where $N_{i}$ is the cycle number at the ith stress level; ${\bar{N}}_{i}$ and $\alpha_{fi}$ are the scale parameter and the shape parameter for the Weibull distribution, respectively; and $P_{s}\left(N_{i}\right)$ is probability of survival after $N_{i}$ cycles. The values of $\sigma_{0}$ and *k* are determined by linearly fitting the experimental $\log\left(\sigma_{i}\right)-\log\left({\bar{N}}_{i}\right)$. $\alpha_{f}$ is solved using the maximum likelihood estimators equations [16]. Thus, the *S*-N curves with different confidence levels are calculated with Equation (8):

(8) $$\sigma =\sigma_{0}\left\{{\left[-\ln(P_{s}(N))\right]}^{\left(\frac{1}{\alpha_{f}k}\right)}\right\}N^{\left(-\frac{1}{k}\right)}.$$

The calculated results with 95% confidence levels are listed in Table 3. It can be seen from Table 3 for the stress ranges of 200 MPa and 400 MPa, the results calculated using NLD method and Whitney’s method were quite close. For higher stress ranges (i.e., 600 MPa and 800 MPa), the predicted results using NLD method were slightly larger. Despite the relative simplicity of NLD method, it assumed 15% COV is typically lower than the experimental results for CFRP, making the predictions less confident. In Whitney’s method, the COV is determined on the basis of the experiments and was thus more suitable for reflecting the variability of the material properties of FRP material [16].

According to the data in Table 3, the equation describing the relationship between the stress range and the maximum stress at 2 million times of fatigue life of CFRP tendons was established in this paper, as shown in Equation (9):

(9) $$\Delta \sigma =1350-\sigma_{\max}.$$

On the basis of either one of the stress ranges and the maximum stress, the other one can be calculated through Equation (9). When the actual loading value was less than the calculated value, the fatigue life of CFRP tendons could be more than 2 million times.

## 4. Conclusions

The fatigue life of CFRP tendons was studied through fatigue test and analysis, and the fatigue performance of the new wedge-type anchorage was verified in this paper. The main conclusions are shown as follows:

(1) In the fatigue cycle, CFRP tendons exhibited burst rupture in the middle portion. The new wedge-type anchorage presented excellent fatigue resistance without tendon slippage or tendon failure at anchorage.
(2) The stress range Δσ and the maximum stress $\sigma_{max}$ were two key parameters affecting the fatigue life N of CFRP tendons. At the same stress range, the greater the maximum stress, the shorter the fatigue life of CFRP tendons. At the same maximum stress, the greater the stress range, the shorter the fatigue life of CFRP tendons.
(3) The bilinear equation and simplified equation for predicting the fatigue life of CFRP tendons established in this paper considered the effects of the stress range Δσ and the maximum stress $\sigma_{max}$. Both were able to provide accurate predictions. The bilinear equation had a higher level of accuracy, whereas the simplified equation was simpler and easy to use.
(4) The predictions obtained using the Whitney’s method suggested that at the 95% confidence level, the CFRP tendons were able to be subjected to 2 million cyclic loads without fatigue failure when the maximum stresses were 63.9%, 53.0%, and 36.8% $f_{u}$ for the stress range of 200 MPa, 400 MPa, 600 MPa, respectively.

In particular, the application scope for the conclusions and equations of this paper were that the stress range was 200–800 MPa and the maximum stress was 0.37–1.0 $f_{u}$ for the fatigue life of CFRP tendons.

## Figures and Tables

**Table 1:** Material properties.

Specimen No.	Dimension		Tensile Properties	
	Length L (mm)	Diameter D (mm)	Strength $f_{fu}\;(\mathrm{Mpa})$	Modulus $E_{f}\;(\mathrm{Gpa})$
1	500	8	2211	146
2			2071	152
3			2038	154
4			2173	147
5			2186	152
Mean			2136	150.2
Dispersion			3.2%	2.1%

**Table 2:** Test results.

Specimen No.	$\sigma_{max}$ (Mpa)	Δσ (Mpa)	N	Fatigue Data	Specimen No.	$\sigma_{max}$ (Mpa)	Δσ (Mpa)	N	Fatigue Data
1	1800	600	296	Mean: 541 SD: 267.5	17	1800	800	27	Mean: 99 SD: 69.5
2	1800	600	913		18	1800	800	77	
3	1800	600	414		19	1800	800	193	
4	1600	600	715	Mean: 1002 SD: 470.2	20	1600	800	382	Mean: 411 SD: 226.7
5	1600	600	626		21	1600	800	149	
6	1600	600	1665		22	1600	800	702	
7	1400	600	3790	Mean: 3736 SD: 1381.2	23	1400	800	870	Mean: 1072 SD: 534.7
8	1400	600	2018		24	1400	800	542	
9	1400	600	5400		25	1400	800	1804	
10	1200	600	11,042	Mean: 16,917 SD: 7199.5	26	1200	800	5105	Mean: 6269 SD: 2732.4
11	1200	600	27,056		27	1200	800	3660	
12	1200	600	12,653		28	1200	800	10,042	
13	1000	600	303,050	Mean: 478,313 SD: 171,393.4	29	1000	800	47,056	Mean: 55,819 SD: 18,275.9
14	1000	600	710,947		30	1000	800	39,143	
15	1000	600	420,942		31	1000	800	81,258	
16	800	600	2,000,000	Mean: 2,000,000 SD: -	32 33 34	810 810 810	800 800 800	1,206,743 831,250 648,311	Mean: 895,435 SD: 232,452.6

SD is standard deviation.

**Table 3:** Comparison of prediction results (units: MPa).

Stress Range	Normal Lifetime Distribution (NLD) Prediction Results	Whitney’s Prediction Results	Prediction Results Equation (3)	Prediction Results Equation (4)
200	1270 (0.635 $f_{u}$)	1277 (0.639 $f_{u}$)	1382 (0.691 $f_{u}$)	1343 (0.672 $f_{u}$)
400	1062 (0.531 $f_{u}$)	1059 (0.530 $f_{u}$)	1051 (0.526 $f_{u}$)	1111 (0.556 $f_{u}$)
600	783 (0.387 $f_{u}$)	745 (0.368 $f_{u}$)	920 (0.455 $f_{u}$)	879 (0.434 $f_{u}$)
800	692 (0.342 $f_{u}$)	648 (0.320 $f_{u}$)	689 (0.340 $f_{u}$)	647 (0.320 $f_{u}$)

## Supplementary Materials

The following are available online at https://www.mdpi.com/1996-1944/12/20/3383/s1, Table S1. Changing rate of fatigue life. Table S2. Comparison between calculation results of simplified Equation (3) and test results. Table S3. Comparison between calculation results of simplified Equation (4) and test results.

## References

[1] Buyukozturk O., Hearing B. (1998). Failure behavior of precracked concrete beams retrofitted with FRP. J. Compos. Constr..

[2] Cao S., Wang X., Wu Z. (2011). Evaluation and prediction oftemperature-dependent tensile strength of unidirectional CFRP composites. J. Reinf. Plast. Compos..

[3] Triantafillou T.C. (1998). Shear strengthening of reinforced concretebeams using epoxy-bonded FRP composites. ACI Struct. J..

[4] Curtis P.T. (1989). The fatigue behaviour of fibrous composite materials. J. Strain. Anal. Eng..

[5] American Concrete Institute (2017). Guide for the Design and Construction of Externally Bonded FRP Systems for Strengthening Concrete Structures.

[6] Noël M., Soudki K. (2014). Fatigue Behavior of GFRP Reinforcing Bars in Air and in Concrete. J. Compos. Constr..

[7] Wu Z., Wang X., Iwashita K., Sasaki T., Hamaguchi Y. (2010). Tensile fatigue behaviour of FRP and hybrid FRP sheets. Compos. Part. B Eng..

[8] Wang X., Shi J., Liu J., Yang L., Wu Z. (2014). Creep behavior ofbasalt fiber reinforced polymer tendons for prestressing application. Mater. Des..

[9] Saadatmanesh H., Tannous F.E. (1999). Long-term behavior ofaramid fiber reinforced plastic (AFRP) tendons. ACI Mater. J..

[10] American Concrete Institute (2004). Prestressing Concrete Structures with FRP Tendons.

[11] Dong Y., Zhang J., Song S., Zhou F., Wang C. (2018). Experimental Investigation on the Creep Property of Carbon Fiber Reinforced Polymer Tendons under High Stress Levels. Materials.

[12] Saadatmanesh H., Tannous F.E. (1999). Relaxation, creep, and fatigue behavior of carbon fiber reinforced plastic tendons. Aci Mater. J..

[13] Adimi M.R., Rahman A.H., Benmokrane B. (2000). New method for testing fiber-reinforced polymer rods under fatigue. J. Compos. Constr..

[14] Zhang X., Ou J. (2006). Experimental study on fatigue behavior of CFRP bars. Chin. J. Mater. Res..

[15] Feng B., Wang X., Wu Z. (2019). Fatigue life assessment of FRP cable for long-span cable-stayed bridge. Compos. Struct..

[16] Vassilopoulos A.P., Keller T. (2011). Fatigue of Fiber-Reinforced Composites.

[17] Practice, Standard ASTM (2010). E739-10 Standard Practice for Statistical Analysis of Linear or Linearized Stress Life (S-N) and Strain Life (ε-N) Fatigue Data.

[18] Zureick A.H., Bennett R.M., Ellingwood B.R. (2006). Statistical characterization of fiber-reinforced polymer composite material properties for structural design. J. Struct. Eng..

[19] Wang X., Shi J., Wu Z., Zhu Z. (2016). Fatigue Behavior of Basalt Fiber-Reinforced Polymer Tendons for Prestressing Applications. J. Compos. Constr..

[20] Xie G.H., Tang Y.S., Wang C.M., Li S.Q., Liu R.G. (2018). Experimental study on fatigue performance of adhesively bonded anchorage system for CFRP tendons. Compos. Part. B Eng..

[21] Wang L., Zhang J., Xu J., Han Q. (2018). Anchorage systems of CFRP cables in cable structures—A review. Constr. Build. Mater..

[22] Sayed-Ahmed E.Y., Shrive N.G. (1998). A new steel anchorage system for post-tensioning application using carbon fiber reinforced plastic tendons. Can. J. Civ. Eng..

[23] Al-Mayah A., Soudki K.A., Plumtree A. (2001). Experimental and analytical investigation of a stainless steel anchorage for CFRP prestressing tendons. PCI J..

[24] Al-Mayah A., Soudki K., Plumtree A. (2006). Development and assessment of a new CFRP rod-anchor system for prestressed concrete. Appl. Compos. Mater..

[25] Schmidt J.W., Bennitz A., Täljsten B., Goltermann P., Pedersen H. (2012). Mechanical anchorage of FRP tendons—A literature review. Constr. Build. Mater..

[26] Nanni A., Bakis C.E., O’Neil E.F., Dixon T.O. (1996). Short-term sustained loading of FRP tendon anchor systems. Constr. Build. Mater..

[27] Zhang X. (2015). Experimental Study on the Fatigue Behavior of Prestressing CFRP Tendon-Anchorage Assembly. Master’s Thesis.

[28] Barron V., Buggy M., Mckenna N.H. (2001). Frequency effects on the fatigue behaviour on carbon fibre reinforced polymer laminates. J. Mater. Sci..

