# Supplementary material for: Experimental Research and Analysis on Fatigue Life of Carbon Fiber Reinforced Polymer (CFRP) Tendons
Source: Materials (Basel). 2019 Oct 16;12(20):3383. doi: 10.3390/ma12203383 (PMC6829243; doi:10.3390/ma12203383)
Supplement: Supplementary file 1 [file materials-12-03383-s001.pdf]

materials

MDPI

Article

Experimental Research and Analysis on Fatigue Life of Carbon Fiber Reinforced Polymer (CFRP) Tendons

Shoutan Song [1,2,*], Hua Zang [2,3], Ning Duan [1] and Juntao Jiang [1]

[1] School of Civil Engineering, Southeast University, Nanjing 210096, China; songshoutan@seu.edu.cn (S.S.); duan_n@outlook.com (N.D.); 220171055@seu.edu.cn (J.J.)
[2] Key Laboratory of Concrete and Prestressed Concrete Structures Ministry of Education, Southeast University, Nanjing 210096, China； zanghua2003@163.com (H.Z.)
[3] School of Architecture Engineering, Nanjing Institute of Technology, Nanjing 211167, China
* Correspondence: songshoutan@seu.edu.cn; Tel.: +86-136-0516-7197

Table S1. Changing Rate of Fatigue Life.

Stress range（Mpa）	K1	K2
200	248.5	-
400	532.9	90.4
600	476.6	189.8
800	386.6	205.4

Table S2. Comparison between Calculation Results of Simplified Equation (3) and Test Results.

Saadatmanesh. 1999				Test data			
Maximum stress (Mpa)	Stress range (Mpa)	Test	Equation (3) Calculation	Maximum stress (Mpa)	Stress range（Mpa）	Test	Equation (3) Calculation
2000	200	4.060	3.796	1800	600	2.733	2.605
1800	200	4.592	4.612	1600	600	3.001	3.067
1600	200	5.276	5.429	1400	600	3.572	3.529
1400	200	6.475	6.245	1200	600	4.228	4.527
2000	400	2.948	2.740	1000	600	5.680	5.797
1800	400	3.431	3.330	800	600	6.301	7.067
1600	400	3.825	3.920	1800	800	1.996	2.139
1400	400	4.074	4.510	1600	800	2.614	2.518
1200	400	6.288	5.851	1400	800	3.030	2.898
				1200	800	3.797	3.841
				1000	800	4.747	4.805
				810	800	5.952	5.720

Table S3. Comparison between Calculation Results of Simplified Equation (4) and Test Results.

Saadatmanesh. 1999				Test data			
Maximum stress (Mpa)	Stress range (Mpa)	Test	Equation (4) Calculation	Maximum stress (Mpa)	Stress range (Mpa)	Test	Equation (4) Calculation
2000	200	4.060	3.459	1800	600	2.733	2.316
1800	200	4.592	4.325	1600	600	3.001	3.182
1600	200	5.276	5.190	1400	600	3.572	4.048
1400	200	6.475	6.056	1200	600	4.228	4.913
2000	400	2.948	2.455	1000	600	5.680	5.779
1800	400	3.431	3.320	800	600	6.301	6.645
1600	400	3.825	4.186	1800	800	1.996	1.312
1400	400	4.074	5.052	1600	800	2.614	2.177
1200	400	6.288	5.918	1400	800	3.030	3.043

	1200	800	3.797	3.909
	1000	800	4.747	4.775
	810	800	5.952	5.597

© 2019 by the authors. Submitted for possible open access publication under the terms and conditions of the Creative Commons Attribution (CC BY) license (http://creativecommons.org/licenses/by/4.0/).
